# Optimizing green approach to enhanced antioxidants from Thai pigmented rice bran using deep eutectic solvent-based ultrasonic-assisted extraction

**DOI:** 10.1016/j.heliyon.2023.e23525

**Published:** 2023-12-13

**Authors:** Pacharawan Ratanasongtham, Wasitthi Bunmusik, Suwaporn Luangkamin, Sugunya Mahatheeranont, Panawan Suttiarporn

**Affiliations:** a Faculty of Science and Technology, Valaya Alongkorn Rajabhat University under the Royal Patronage, Pathum Thani, 13180, Thailand; b Faculty of Science, Energy and Environment, King Mongkut's University of Technology North Bangkok, Rayong Campus, Rayong, 21120, Thailand; c Department of Fundamental Science and Physical Education, Faculty of Science at Sriracha, Kasetsart University, Sriracha Campus, Chonburi, 20230, Thailand; d Department of Chemistry, Faculty of Science, Chiang Mai University, Chiang Mai, 50200, Thailand

**Keywords:** Deep eutectic solvent, Green extraction, Thai pigmented rice bran, Antioxidant compounds

## Abstract

Deep eutectic solvents (DES) have garnered significant attention as extraction media owing to their commendable attributes of being environmentally sustainable and the inherent adaptability of DES's versatile physical and chemical characteristics. The present study investigated the effects of deep eutectic solvents on the total contents of anthocyanin, phenolic, and flavonoids, as well as the antioxidant activity of Thai pigmented rice bran extract. The optimal extraction parameters for deep eutectic solvent-based ultrasonic-assisted extraction (DES-UAE) were also determined using the response surface methodology (RSM). The optimal conditions for the extraction of anthocyanins and other antioxidants from pigmented rice bran using a deep eutectic solvent were choline chloride: ethylene glycol (Ch:Eg) at a 1:2 ratio, mixed with 20 % water as a solvent. The ultrasonic-assisted extraction (UAE) at 37 kHz of frequency, 50 °C of temperature, 40 min of extraction time, and a 1:6 g/mL of solid-to-solvent ratio yielded a total anthocyanin content of 4.55 ± 0.09 mg C3G/g DW, a total phenolic content of 26.49 ± 0.62 mg GAE/g DW, a total flavonoid content of 6.57 ± 0.55 mg QE/g DW, and a percent inhibition of DPPH radical of 77.83 ± 1.51. By comparing the antioxidant content that was extracted from three cultivars of pigmented rice, it was found that Leum Pua black rice bran provided significantly higher antioxidant content compared to Hom Nin purple rice bran and Mali Dang red rice bran. This research suggests an achievable, eco-friendly, and effective method for preparing high-quality, consumer-safe Thai rice bran as a raw material for nutraceuticals.

## 1 Introduction

Pigmented rice cultivars, whose bran colors vary from white to brown, red, dark purple, and black, are being increasingly consumed because of their health-promoting potential and chemopreventive efficacy [1]. Rice by-products, including husk, bran, and germ, from rice milling processes have been identified as a rich source of numerous bioactive compounds, particularly pigmented rice bran. Pigmented rice bran contains very high nutritional content and abundant bioactive phytochemicals such as phenolic compounds, phytosterols, tocopherol, proanthocyanin, and anthocyanin, which exhibit greater antioxidant activity than bran from non-pigmented rice [2,3]. A number of health-promoting benefits, including a decreased risk of chronic illnesses, have been attributed to biologically active compounds, which include phenolic componds [4]. Anthocyanin and proanthocyanidin are the principal color compounds that can resist the oxidative stress caused by diverse intermediates to protect cells from cellular damage [5]. Flavonoids exhibit a strong antioxidant capacity, lower blood sugar and cholesterol levels, lessen the risk of cardiovascular disease, and prevent the absorption of cholesterol in humans [6].

Conventional processes such as maceration, refluxing, Soxhlet extraction, and solid-liquid extraction, which usually employ suitable and efficient solvents (ethanol, methanol, acetone, and water), are the most widely used techniques for obtaining antioxidant phytochemical components from plants. The optimal extraction method is crucial to achieve high efficiency, purity, and maximum content of bioactive compounds. Additionally, eco-friendly solvents should be selected for the extraction method. The green technique that enhances extraction efficiency, such as solvent ultrasonic-assisted extraction (UAE), has attracted interest as a potential alternative method to conventional extraction techniques as it can enhance the mass transfer rate within the solution. The UAE has the benefit of requiring shorter extraction times, reducing solvent consumption, and operating at lower temperatures, which is good for the extraction of thermally sensitive anthocyanins [7,8]. Based on the type of solvent and its level of concentration, the UAE method's effectiveness varies.

A green alternative, deep eutectic solvent (DES), is one of the challenging extraction solvents produced by hydrogen bond interaction between hydrogen bond acceptors and donors and is a novel type of ionic liquid system. In comparison to organic solvents, DES is regarded as an eco-friendly and efficient extraction solvent due to its stability, low volatility, ease of synthesis, and degradability [9]. DES based on choline chloride as HBA in the combination with different HBD are the most commonly used green solvents for the extraction of value-added compounds from different matrices. The use of organic acid-based NADES demonstrated a high extraction yield of *H. sabdariffa*'s bioactive components and a remarkable selectivity for anthocyanins in particular [10]. Using polyalcohol-based DES is the most effective way to combine DES with UAE for the extraction of various flavonoid subclasses [11]. Choline chloride-fructose natural deep eutectic solvent (NADES) was employed for the extraction of chokeberry, resulting in an extract with the highest concentrations of total phenols and total flavonoids [12]. In addition to the solvent's inherent qualities, choline chloride:urea (1:2) gave rise to high yields of hydrolyzable tannins, gallic acid, and ellagic acid in *Alchemilla vulgaris* L [13]. Due to DES's polarity being similar to that of water and highly polar organic solvents and solvent penetration that is aided by ultrasound's capillary effects, the extraction efficiency of antioxidants has been enhanced when deep eutectic (DES) solvents are combined with UAE [14].

The efficiency of the recovery of bioactive chemicals from natural resources is not only influenced by the type of DES but also by UAE time, temperature, and the material-to-solvent ratio. Therefore, DES-UAE conducted an optimization without using appropriate statistical methods, namely response surface methodology (RSM), that are essential for achieving the maximum yield of the targeted compound. Nevertheless, there have been no reports on the optimal conditions of the deep eutectic solvent-based ultrasonic-assisted extraction (DES-UAE) technique to simultaneously reach the highest extraction yield of anthocyanins, phenolics, flavonoids, and antioxidant activity from Thai pigmented rice bran. The present study used environmentally friendly DES and UAE for recovery of the antioxidant compounds from Thai pigmented rice bran. The optimal DES-UAE conditions for achieving the maximum content of antioxidant compounds, including the effect of DES type, water content, UAE time, and solid-to-DES ratio on bioactive compounds (total anthocyanin content; TAC, total phenolic content; TPC, and total flavonoid content; TFC) and antioxidant activities were evaluated using RSM with a central composite design (CCD). Moreover, the optimized DES-UAE process was used to compare the antioxidant content of different Thai pigmented rice bran cultivars.

## 2 Materials and methods

### 2.1 Chemicals and reagents

2-Diphenyl-1-picrylhydrazyl (DPPH), sodium acetate and quercetin were provided from Sigma Aldrich Chemical Co. (St Louis, MO, USA). Aluminium chloride, citric acid (≥99.7 %), ethylene glycol (≥99.5 %), fructose, glycerol, oxalic acid, urea (≥99 %), and sodium carbonate were taken from Kemaus (New South Wales, Australia). Folin-Ciocalteu's reagent and choline chloride (≥98 %) were obtained from Loba Chemie Pvt. Ltd. (Mumbai, India). Methanol (AR grade) and ethanol (AR grade) were purchased from RCI Labscan Ltd. (Bangkok, Thailand). Gallic acid (>99 %) was acquired from Merck Co. (Weiterstadt, Germany).

### 2.2 Rice materials

Three samples of rice (*Oryza sativa* L.), Leum Pua, Homnin and Mali Dang, were used in this study. Only Leum Pua was used in the optimization experiment. A pigmented glutinous rice variety Leum Pua was provided by the Phitsanulok Rice Research Center in Thailand's Phitsanulok province. Homnin and Mali Dang were provided by Salana Organic Village (social enterprise) Company Limited, Thailand's Nakhon Pathom province. After being harvested, freshly cultivated rice paddies were exposed to the sun until their moisture level dropped to less than 14 %. Rice grains were processed using local milling technology after being dehusked. Before being used in experiments, the bran samples were sieved through a 45 mesh screen, packed in a plastic bag with less pressure, and stored at 4 °C.

### 2.3 Preparation of deep eutectic solvents (DES)

The DESs were synthesized by mixing the HBA (choline chloride; Ch) and HBD (oxalic acid; Oa, glycerol; Gy, ethylene glycol; Eg, urea; Ur and fructose; Fu) at various ratios. The HBA and HBD were mixed under magnetic stirring and heating at 80 °C until a clear homogeneous liquid of deep eutectic solvent was obtained. The water was added (based on the experiment conditions) to DES in order to reduce the viscosity before extraction. The type of DES and mixing ratio were shown in Table 1. The physicochemical characteristics of DES including pH and viscosity were investigated. The pH of DES was measured using pH meter (Model FE28 FiveEasy, Mettler, Switzerland). In addition, the Cannon-Fenske-type viscometer (CANNON, PA, USA) has been gradually used for the measurement of liquid viscosity. A thermostatically controlled bath is used in the measurements of viscometers to provide thermal equilibrium. The kinematic viscosity was determined at 80 ± 1 °C by multiplying the constant of viscometer tube and the measured efflux time. For each experiment, the measurements were performed three times, and the average result was calculated.

**Table 1:** List of DES for extraction.

Code	Type of DES	Ratio	Code	Type of DES	Ratio
**D1**	Ch:Oa	1:0.5	D2	Ch:Oa	1:1
**D3**	Ch:Oa	1:1.5	D4	Ch:Gy	1:1
**D5**	Ch:Gy	1:2	D6	Ch:Gy	1:3
**D7**	Ch:Eg	1:1	D8	Ch:Eg	1:2
**D9**	Ch:Eg	1:3	D10	Ch:Ur	1:0.5
**D11**	Ch:Ur	1:1	D12	Ch:Ur	1:1.5
**D13**	Ch:Fu	1:0.5	D14	Ch:Fu	1:1
**D15**	Ch:Fu	1:1.5			

### 2.4 Ultrasonic-assisted extraction (UAE)

The antioxidant extraction from glutinous pigmented rice bran (Leum Pau) was performed as follows: 1.00 g of rice brans and 5.0 mL of different ts of DES (D1-D15) (30 % water content and 1:5 g/mL rice bran-to-DES ratio) and 60 % (v/v) aqueous methanol with 0.1 % citric acid (conventional solvent, 1:5 g/mL) were extracted using an ultrasonic bath (Elmasonic P 300, Elma, Germany) at 50 °C and 37 kHz. Three cultivars of Thai pigmented rice bran, Leum Pau, Homnin, and Mali Dang, were extracted under the optimum conditions of DES-UAE and conventional extraction. The extracted solution was then filtered through filter paper No. 1, and methanol was added to the supernatant in order to obtain a volume of 10 mL. The supernatant was kept in amber glass bottles and stored in the refrigerator before TAC, TPC, TFC, and DPPH assay analysis.

### 2.5 Determination of antioxidant content and activity in the DES rice bran extracts

#### 2.5.1 Total anthocyanin contents (TAC)

The pH differential technique, which was significantly modified from the Giusti and Wrolstad method, was applied to investigate the TAC [15]. The 25 μL of rice bran extract was diluted to 100 μL with buffer solution (pH 1.0 or pH 4.5) in the microplate. Following 30 min of dark, room temperature incubation of the test solution, the absorbance at 520 and 700 nm was determined using a microplate reader (M956, Metertech, Taiwan). Equation (1) was used to calculate the TAC, which was then expressed in mg C3G/g DW.

(1) $$\text{TAC}\;\left(\text{mg}\;C3G/g\;\text{DW}\right)=\frac{A\times \text{MW}\times \text{DF}}{\varepsilon \times L}\times \frac{V}{m}$$

where A was the absorbance of the test solution at 520 and 700 nm; A = (A_520nm_ –

A_700nm_) pH 1.0 – (A_520nm_ – A_700nm_) pH 4.5; MW the was the molecular weight of cyaniding-3-glucoside (C3G) = 449.2 g/mol; DF was dilution factor of sample; ε was molar absorptivity = 26,900 L/mol.cm; L was the path length of the cuvette = 1 cm, V was the volume of extract (mL), and m was the weight of the sample.

#### 2.5.2 Total phenolic content (TPC)

The total phenolic content (TPC) was investigated by Folin Ciocalteu method modified from Thitilertdecha et al. [16]. The DES rice bran extract (25 μL) and Folin reagent (100 μL) were mixed in microplates. Then, sodium carbonate solution (75 μL) was added, and the resulting mixture was left at room temperature in the dark for 2 h. The absorbance of the mixture solution was investigated at 765 nm using a microplate reader. A calibration curve was prepared using gallic acid as the standard. The TPC of DES extracts was calculated by comparing them to the gallic acid calibration curve and exhibited as milligrams of gallic acid equivalents per gram of rice bran dry weight (mg GAE/g DW).

#### 2.5.3 Total flavonoid content (TFC)

Total flavonoid content (TFC) was evaluated by the aluminium chloride colorimetric method modified from Sembiring et al. method [17]. A 50 μL rice bran extract and 150 μL methanol were added to a microplate with 10 μL sodium acetate. Then, 10 μL of aluminum chloride was added and left at room temperature in the dark for 40 min. The absorbance of the solution was assessed at 415 nm using a microplate reader. The quercetin calibration curve was used to determine the TFC, which was then reported as milligrams of quercetin equivalents per gram of dry weight rice bran (mg QE/g DW).

#### 2.5.4 Antioxidant assays (DPPH radical scavenging assay)

The antioxidant activity of the pigmented rice bran extract was investigated by DPPH radical scavenging assay slightly modified from Sembiring et al. method [17]. The rice bran extract (20 μL) was added to the ethanolic DPPH solution (180 μL) in a microplate and then allowed at room temperature in the dark for 30 min. Additionally, a control sample was generated, which comprised the DPPH solution without the sample. The DPPH solution was replaced with ethanol for the blank solution [18]. The absorbance of the solution was investigated at 517 nm using a microplate reader. The percentage of DPPH inhibition was calculated by the following equation.

(2) $$\text{DPPH}\;\text{radical}\;\text{scavenging}\;\text{activity}=\frac{A_{c}‐A_{s}}{A_{c}}\times 100$$

where A_c_ was the absorbance of the control and A_s_ was the absorbance of the sample.

### 2.6 The effect of a certain factor on antioxidant contents and activitiess

The influence of a single factor on the antioxidant contents and activities of pigmented rice bran and the optimal range for each single factor were ascertained by a single-factor experiment. The rice bran was extracted by UAE with an appropriate deep eutectic solvent (Ch:Eg; 1:2). The parameters, including water content (0–50 %), extraction time (30–50 min), and solid-to-solvent ratio (1:2–1:10 g/mL), were all examined. A single-factor investigation was performed to study the impact of different variables on the response, including TAC, TPC, TFC, and antioxidant activity.

### 2.7 Design of experiment

RSM, incorporated with CCD, was used to construct a statistical model for the antioxidant separation from pigmented rice bran with UAE. The central composite design (CCD) was applied to perform optimization experiments with a total of 20 experimental runs. The suitable ranges of water content (X_1_), extraction time (X_2_), and solid-to-solvent ratio (X_3_) for the antioxidant content and activity were achieved based on a single-factor experiment. Each DES-UAE independent variable consisted of 3 levels ranging from low (−1), to medium (0), and to high (+1). The factors of CCD design were employed to determine the total anthocyanin content (Y_1_), total phenolic content (Y_2_), total flavonoid content (Y_3_), and DPPH radical scavenging activity (Y_4_). The levels of the independent factor anddependent variables of DES extraction were shown in Table 2.

**Table 2:** Code levels of the independent and factor of dependent variables of DES extraction.

Symbol	Independent variables	Units		Code levels		Symbol	Independent variables	Units
			−1	0	1			
X_1_	Water content	%	20	30	40	Y1	Total anthocyanin content	mgC3G/g DW
X_2_	Extraction time	C	30	35	40	Y2	Total phenolic content	mg GAE/g DW
X_3_	Solid-to-solvent ratio	g/mL	1:2	1:4	1:6	Y3	Total flavonoid content	Mg QE/g DW
						Y4	Percent inhibition of DPPH	%

The design of experiment consisted of 20 runs, including 6 axial experiments, 8 factorial experiments, and 6 replicates in central point to estimate pure error and assess the lack of fit of the proposed models. After obtaining the data, RSM was used to determine the optimal processing setting for each independent variable. The statistical significance of the model was systematically evaluated using ANOVA and RSM analysis. The optimal conditions of DES-UAE of antioxidant compounds and antioxidant activity were investigated using a second-order polynomial model (Equation (3)). The model fitting was applied based on coefficient (R^2^), adjusted determination coefficient (R^2^adj), predicted-R-squared, and the lack-of-fitness statistic [19].

(3) $$Y=\beta_{0}+\sum_{i=1}^{3}\beta_{i}X_{i}+\sum_{i=1}^{3}\beta_{ii}X_{i}^{2}+\sum_{i=1}^{2}\sum_{j=i+1}^{3}\beta_{ij}X_{i}X_{j}+\varepsilon$$

where Y is the dependent antioxidant responses, X_i_ is the independent factor, β_0_, β_i_, β_ii_ and β_ij_ are the regression coefficients of the intercept, linear, quadratic and interaction terms, and “***ε***” is the random error.

### 2.8 Statistical analysis

The values of all responses were presented as mean ± standard deviation. Statistical analysis involved the use of ANOVA, and significant differences (p < 0.05) among the means were determined using Duncan's multiple comparison test. The two-sample T-test was employed to analyze the significant differences (p < 0.05) for a comparative study of extraction techniques. The statistical analysis was performed using Minitab statistical software (Trial version 18, Minitab Inc., State College, PA, USA).

## 3 Results and discussion

### 3.1 Physical properties of DES

The modification of HBD type and/or the molar ratio has the potential to impact the physicochemical properties, including viscosity and pH [20]. Four DES groups, including organic acid-based, alcohol-based, amide-based, and sugar-based, were examined in this experiment. Organic acid-based DES may provide a system with different acidity and basicity. Alcohol-based DES, using alcohols like glycerol or ethylene glycol, may offer a different polarity. Urea functions as a hydrogen bond donor, hence there may be differences in the way amide-based DES interacts with target compounds. DES using sugars, such as fructose, could provide a hydrophilic environment. The physicochemical characteristics of DES including pH and viscosity were displayed in Table 3.

**Table 3:** Physicochemical characteristics of DES.

Code	Type of DES (30 % water)	Ratio	Characteristic of DES	
			pH	Viscosity mm^2^/s
D1	Ch:Oa	1:0.5	<1	8.21
D2	Ch:Oa	1:1	<1	6.27
D3	Ch:Oa	1:1.5	<1	5.99
D4	Ch:Gy	1:1	5.10	10.37
D5	Ch:Gy	1:2	5.28	11.00
D6	Ch:Gy	1:3	5.37	11.33
D7	Ch:Eg	1:1	4.91	5.94
D8	Ch:Eg	1:2	5.00	5.49
D9	Ch:Eg	1:3	5.13	5.20
D10	Ch:Ur	1:0.5	7.11	6.16
D11	Ch:Ur	1:1	7.08	5.35
D12	Ch:Ur	1:1.5	7.35	4.07
D13	Ch:Fu	1:0.5	3.39	11.86
D14	Ch:Fu	1:1	4.12	23.91
D15	Ch:Fu	1:1.5	4.15	31.80

The viscosity of DES played a significant role in the extraction of phenolic compounds. The composition of DES as well as choline chloride concentration caused considerable differences in their viscosity. The investigated DESs' viscosity ranged from 4.07 to 31.80 mm^2^/s. Viscosity of DES (30 % water, at 1:1 ratio of HBA:HBD) is ordered as follows: sugar-based DES > alcohol (Gy)-based DES > organic acid-based DES > alcohol (Eg)-based DES > amide-based DES. The viscosity indicated that D12 was the least viscous while D15 had the highest viscosity value. The viscosity could be related to the hydrogen bonds that exist between HBA and HBD [21]. Highly viscous solvents limit the diffusion of phenolic compounds from the plant matrix by obstructing molecular mobility within the solvent [22].

The pH behavior of a DES could be influenced by the ionization characteristics of its components. The pH behavior of the DES was investigated at room temperature, and the results are given in Table 3. The acidity of the resulting mixture may be influenced by the sort of hydrogen bond donor (HBD). When HBD is dissolved in a solvent, it may donate a proton (H^+^) and become ionized, affecting the pH of the solution. The organic acid-based DES was shown to possess the lowest pH (<1) followed by sugar-based DES (3.39–4.15), alcohol-based DES (4.91–5.37), and amide-based DES (7.08–7.35). The hydrogen-bond donor of organic acid has a strong effect on the resultant pH. The lower pH than 7 of alcohol-based DES was caused by the presence of alcohol, which has acidic hydrogen in its structures [23]. The presence of the NH bonds may contribute to a mild acidity in the amide-based DES. The ionization tendency of NH bonds in urea is generally weaker compared to strong acids.

### 3.2 Assessment of DES extraction effectiveness

**Fig. 1:**
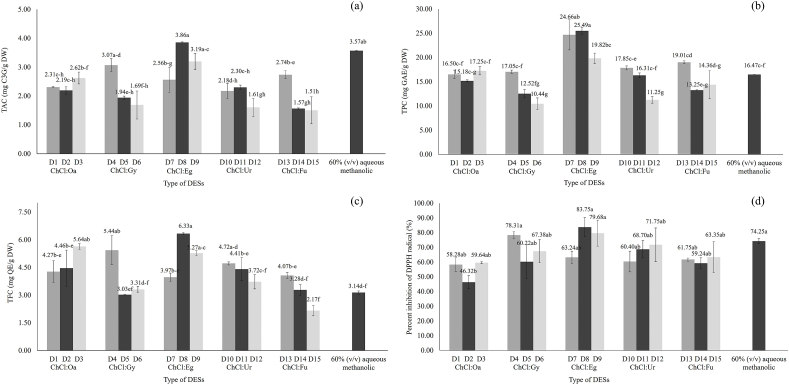
(A)TAC; (b) TPC; (c) TFC); and (d) DPPH assay of pigmented rice bran extracts extracted with 15 different DES types and conventional solvent. Significant differences between samples were indicated by different letters on the bars (p < 0.05). The value (n = 3) demonstrates the mean ± SD.

The various types of DES (Table 1) were used for antioxidant extraction from brans of pigmented rice using ultrasonic-assist extraction (30 % water content, 1:5 g/mL rice brans-to-DES ratio, and 37 kHz) at 50 °C for 35 min. The selection of DES type was the essential parameter in antioxidant extraction because the extraction efficiency was observed to be dependent on the viscosity and polarity of DES. The antioxidant contents (TAC, TPC, and TFC) of the pigmented rice bran extracts extracted with 15 different DES types and their antioxidant capacity were shown in Fig. 1.

Fig. 1 reveals that the optimal DES type for bioactive compound extraction was choline chloride: ethylene glycol (Ch: Eg) 1:2 (D8), which displayed the highest TAC, TPC, TFC and percent inhibition of DPPH radical (3.86 ± 0.02 mg C3G/g DW, 25.49 ± 0.67 mg GAE/g DW, 6.33 ± 0.06 mg QE/g DW and 83.75 ± 6.57 %, respectively). In terms of antioxidant content and activity, the D8 extract derived from choline chloride-based alcohol exhibited significant superior results compared to DES extracts derived from other HBD (organic acids, amides, and sugars) as well as conventional solvents.

The extraction efficiency generally depended on the type of DES, viscosity, solubility, and polarity. As illustrated in Fig. 1, among the 15 types of choline-based DES, the best extraction efficiency of anthocyanin, flavonoids and phenolics was obtained with Ch:Eg (D7-D9), consistent with the previous reports [22,24,25].

The results indicated that the DES with alcohol groups as the HBDs, which displayed strong multiple hydrogen bonding, could establish strong interactions with antioxidant compounds as well as acid groups. The hydrophilicity of phenolic compounds plays a crucial role in enhancing the polarity of deep eutectic solvents through hydrogen bonding and polar interactions. The hydroxyl groups in phenolic compounds can form hydrogen bonds with both the HBD and HBA components of the DES, creating a network of interactions. The collectively enhance the polarity of the deep eutectic solvent (DES), improving its interactions with other substances like sugars, polyalcohols, organic acids, and choline chloride, and thereby facilitating the extraction process. Phenolic compounds are more soluble in the DES when hydrogen bonds are enhanced because dipole-dipole and ion-dipole interactions weaken the forces binding the phenolic component in the plant matrix and facilitate its homogeneous dissolution throughout the DES [22].

However, the performance of the antioxidant compound extraction decreased when utilizing Ch: Oa (D1-D3) as the HBDs. This might be because the high acidity of the solvent caused the decomposition of anthocyanin and reduced extraction efficiency [26]. In addition, the Ch: Oa solvent system demonstrated greater viscosity than the Ch: Eg (D7-D9). The high viscosity of the solvent system might hinder the mass transfer rate and reduce the extraction efficiency [22]. For HBDs containing an amide group (Ch: Ur; D10-D12), the polar amino groups generated weaker intermolecular hydrogen bonds with anthocyanin, phenolics, and flavonoids than the alcohol and acid groups resulting in a decrease in the extraction efficiency of antioxidant compounds. Using Ch: Fu (D13-D15) as HBDs resulted in the lowest extraction activity. Even though fructose was more polar than alcohol groups, its high viscosity impeded the mass transfer of target compounds [27,28]. These extracts' strong antiradical activity may be attributed to their high concentration of phenolic compounds, which have the ability to scavenge radicals. Numerous investigations have shown that the great recovery of bioactive components from DES extracts contributes to their remarkable antiradical activity [29]. Moreover, choline chloride-ethylene glycol was also the most efficient solvent, in agreement with those reported by Wang and Wang (2019) for flavonoids. Therefore, alcohol-based DES (Ch: Eg, 1:2) was selected for the extraction of antioxidant compounds from brans of pigmented rice [11].

### 3.3 Single-factor investigation

Selecting an appropriate range of experimental variables of DES-UAE process contributes to the improvement of the yield of antioxidants from pigmented rice bran. Acquiring an overall understanding of the variables that could impact the procedure is essential. The DES-UAE parameters include water content, UAE time, and rice bran-to-DES ratio, significantly influencing the extraction procedure. The single-factor experiment examines the effects of a single variable while holding all other variables constant. Too-high or too-low experimental factor levels were not conducive to the extraction of bioactive compounds. For a preliminary study, the single-factor method could illustrate the range of factors affecting the response variables.

#### 3.3.1 Effect of water content

**Fig. 2:**
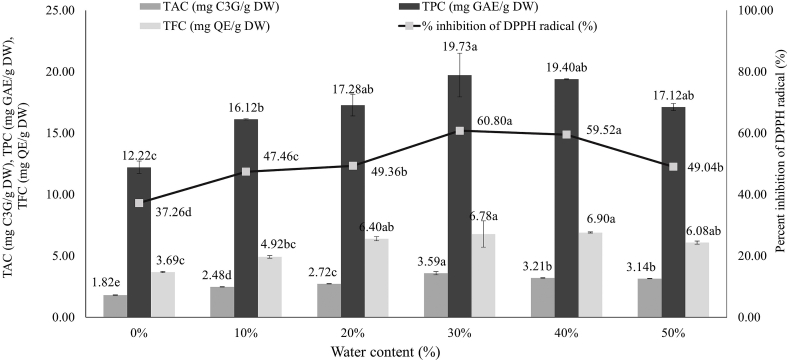
Effect of water content on bioactive compound contained in pigmented rice bran. Significant differences between samples were indicated by different letters on the bars (p < 0.05). The value (n = 3) demonstrates the mean ± SD.

In order to determine the optimum water content for antioxidant extraction, the extraction procedures were performed with different water content (0–50 %). Results indicated that as the water content in the DES solution increased from 0 % to 30 %, an increase in TAC, TPC, and TFC values as well as their antioxidant activity were noted (Fig. 2). At 30 % of water content, there is the highest increase in bioactive compound contents, with a significant difference in TAC. Nevertheless, as the water content went from 30 % to 50 %, the amounts of TAC decreased with significant differences and the amount of TPC as well as their antioxidant activity dramatically decreased. The viscosity of these solvents may be lowered by adding water to the DES system, which would enhance the mass-transfer rate of bioactive chemicals and increase extraction efficiency by reducing the hindrance of mass transfer [30]. Moreover, water can be added to adjust the polarity of the solution. However, higher water content (>30 %) is unfavorable. It will weaken the interactions between the DES and the target compounds. If the water content in the solvent exceeds the limit for forming bonds with the DES components, it disrupts the hydrogen bond of DES and has an adverse effect on the solvent's ability to extract [31]. For extensive evaluation, the range of water content was chosen to be from 20 % to 40 % for further RSM optimization.

#### 3.3.2 Effect of the extraction time

**Fig. 3:**
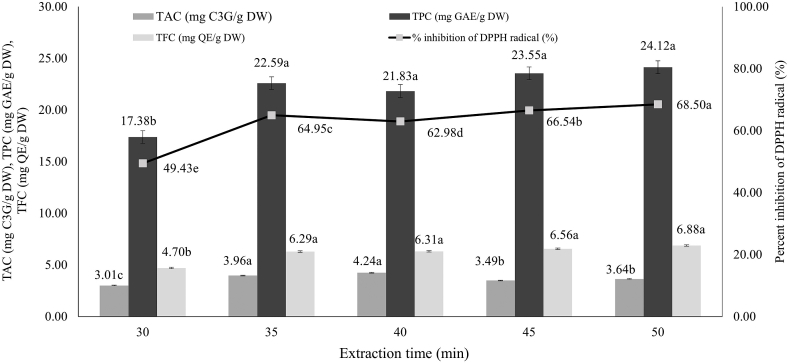
Effect of extraction time on antioxidant compounds contained in pigmented rice bran. Significant differences between samples were indicated by different letters on the bars (p < 0.05). The value (n = 3) demonstrates the mean ± SD.

The effect of UAE time for bioactive compound extraction was investigated at different times (30–50 min). UAE of analytes from solid samples into the liquid phases was performed via a cavitation mechanism [32]. The results from Fig. 3 indicated that the highest TPC, TFC and antioxidant activity in pigmented rice brans was accomplished at 50 min extraction time (water content; 30 %, rice brans-to-solvent ratio; 1:5 g/mL, temperature; 50 °C and frequency; 7 kHz). This is due to the increase of exposure of plant and solvent and helps their release into the solvent. Furthermore, exposure to UAE for a long time can cause structural damage to the solute and decrease the extraction yield. Especially for TAC, the extraction yield remarkably reduced when the extraction time was over 40 min. This could suggest that prolonged extraction times caused the decomposition of anthocyanins [33]. However, there was no significant difference in TPC and TFC when the extraction time increased from 40 to 50 min. As a result, the extraction time in the range of 30–40 min was chosen for future investigations.

#### 3.3.3 Effect of the solid-to-solvent ratio

**Fig. 4:**
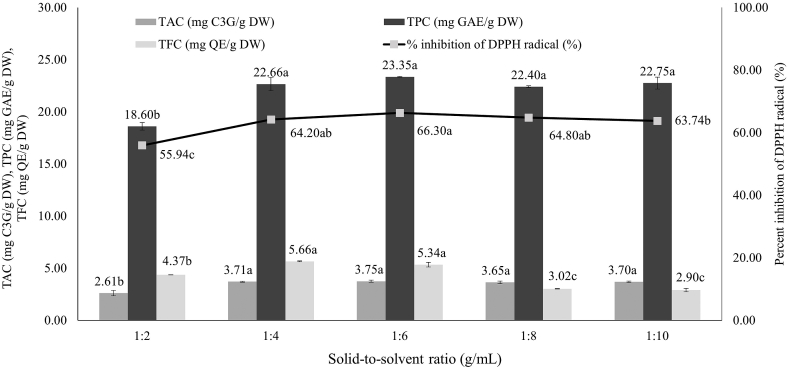
Effect of solid-to-solvent ratio on bioactive compound contained in pigmented rice bran. Significant differences between samples were indicated by different letters on the bars (p < 0.05). The value (n = 3) demonstrates the mean ± SD.

A series of DES-UAE were performed with different rice brans-to-solvent ratios to evaluate the effects of the rice bran-to-DES ratio (1:2, 1:4, 1:6, 1:8, and 1:10 g/mL). As shown in Fig. 4, the antioxidant contents and antioxidant activity of rice bran extracts slightly increased in ratio to 1:6 g/mL. At 1:6 g/mL of rice brans-to-solvent ratio was found that TAC, TPC and antioxidant activity had the highest increased. At ratios of 1:8 and 1:10, TFC began to decrease with significant different when TAC, TPC, and antioxidant activity were not significantly different. The extraction process is not completed when the extraction solvent's volume is insufficient because the sample cannot be fully soaked by the solvent. It is possible to extract the target component into the extraction solvent rapidly when the solvent volume is enormous. Further increasing the solvent volume had no effect on the extraction efficiency [34]. Hence, the solid-to-DES ratio used for the further experiment was within the range of 1:2–1:6.

### 3.4 Fitting model

Based on the single-factor experiment, the acceptable range of conditions needed for extraction of the antioxidant compounds has these three variables, including water content (X_1_), extraction time (X_2_) and solid-to-solvent ratio (X_3_), which were selected for further optimization using RSM. The CCD was then applied to construct 20 experimental designs for RSM randomly; the TAC, TPC, TFC and DPPH radical scavenging activityunder different extraction conditions were shown in Table 4.

**Table 4:** The central composite design (CCD) for optimizing DES extraction with the observed response of each variable.

Run Order	Extraction variables			TAC (mg C3G/g DW)	TPC (mg GAE/g DW)	TFC (mg QE/g DW)	Percent Inhibition of DPPH radical (%)
	X_1_	X_2_	X_3_				
20	0 (30)	0 (35)	0 (1:4)	4.01 ± 0.17	20.97 ± 1.12	5.11 ± 0.27	69.82 ± 0.90
10	1 (40)	0 (35)	0 (1:4)	3.76 ± 0.10	21.78 ± 0.87	5.40 ± 0.34	73.48 ± 0.12
15	0 (30)	0 (35)	0 (1:4)	3.82 ± 0.09	21.55 ± 0.63	5.41 ± 0.29	72.56 ± 0.26
5	−1 (20)	−1 (30)	1 (1:6)	4.12 ± 0.42	26.92 ± 0.30	5.42 ± 0.22	77.41 ± 0.25
12	0 (30)	1 (40)	0 (1:4)	3.88 ± 0.11	22.13 ± 0.48	5.51 ± 0.02	73.53 ± 0.17
6	1 (40)	−1 (30)	1 (1:6)	3.40 ± 0.25	27.37 ± 1.44	6.35 ± 0.12	78.28 ± 0.60
18	0 (30)	0 (35)	0 (1:4)	3.99 ± 0.36	21.33 ± 0.69	5.77 ± 0.13	72.38 ± 0.70
9	−1 (20)	0 (35)	0 (1:4)	4.15 ± 0.09	24.21 ± 1.89	6.17 ± 0.14	74.71 ± 0.71
3	−1 (20)	1 (40)	−1 (1:2)	3.36 ± 0.22	23.50 ± 1.68	6.33 ± 0.09	74.49 ± 0.79
11	0 (30)	−1 (30)	0 (1:4)	4.04 ± 0.18	20.87 ± 1.06	5.41 ± 0.13	69.14 ± 0.62
14	0 (30)	0 (35)	1 (1:6)	3.80 ± 0.09	25.34 ± 0.75	5.38 ± 0.27	75.40 ± 0.84
17	0 (30)	0 (35)	0 (1:4)	4.03 ± 0.45	21.24 ± 1.03	5.56 ± 0.24	70.37 ± 0.43
13	0 (30)	0 (35)	−1 (1:2)	3.35 ± 0.15	17.79 ± 0.75	4.42 ± 0.40	64.24 ± 0.79
19	0 (30)	0 (35)	0 (1:4)	4.07 ± 0.22	21.31 ± 0.88	5.35 ± 0.25	71.28 ± 0.72
1	−1 (20)	−1 (30)	−1 (1:2)	3.65 ± 0.20	19.16 ± 1.53	6.44 ± 0.08	67.53 ± 0.84
2	1 (40)	−1 (30)	−1 (1:2)	3.44 ± 0.09	20.44 ± 0.64	5.36 ± 0.14	68.54 ± 0.42
8	1 (40)	1 (40)	1 (1:6)	3.79 ± 0.10	25.90 ± 0.60	5.84 ± 0.26	76.18 ± 0.34
7	−1 (20)	1 (40)	1 (1:6)	3.96 ± 0.14	31.16 ± 1.95	6.53 ± 0.33	79.06 ± 0.79
16	0 (30)	0 (35)	0 (1:4)	3.99 ± 0.08	20.85 ± 0.33	5.50 ± 0.19	68.77 ± 0.45
4	1 (40)	1 (40)	−1 (1:2)	3.39 ± 0.06	18.56 ± 0.92	3.93 ± 0.58	65.62 ± 0.87

The experimental values varied in the range of 3.35–4.15 mg C3G/g DW for TAC, 17.79–31.16 mg GAE/g DW for TPC, 3.93–6.53 mg QE/g DW for TFC and 64.24–79.06 % for percent inhibition of DPPH radical (Table 4). The quadratic polynomial models were simulated and ANOVA was used to evaluate the adequacy and fitness of the model (Table 5). For ANOVA analysis, the results revealed that the four models were highly significant (P < 0.0001). The values of R^2^, Adj-R^2^ and Pred-R^2^ for TAC (0.9493, 0.9037, and 0.6634, respectively), TPC (0.9975, 0.9953, and 0.9905, respectively), TFC (0.9358, 0.8781, and 0.7383, respectively) and percent inhibition of DPPH radical (0.9271, 0.8614, and 0.4290, respectively) were near to 1, verified that the model was substantial and reasonable. Furthermore, the lack of fit values was not significant (P > 0.05), for TAC (0.538), TPC (0.737), TFC (0.479), and % inhibition (0.444), indicating predictive accuracy of the model for DES extraction [35]. The response variable and test variable can be related using the second-order polynomial equation as displayed in Table 6.

**Table 5:** Analysis of Variance (ANOVA), factors and their interaction effects of DES extraction.

Source	Y_1_: TAC		Y_2_: TPC		Y_3_: TFC		Y_4_: Percent Inhibition of DPPH radical	
	*F*-value	*p*-value	*F*-value	*p*-value	*F*-value	*p*-value	*F*-value	*p*-value
Model	20.80	0.000^a^	450.21	0.000^a^	16.21	0.000^a^	14.12	0.000^a^
X_1_	29.23	0.000^a^	231.73	0.000^a^	32.13	0.000^a^	5.20	0.046^a^
X_2_	1.00	0.341 ^NS^	82.15	0.000^a^	1.41	0.262 ^NS^	2.67	0.133 ^NS^
X_3_	48.47	0.000^a^	2704.86	0.000^a^	18.46	0.002^a^	88.89	0.000^a^
X_1_^2^	0.06	0.807 ^NS^	206.47	0.000^a^	15.32	0.003^a^	11.75	0.006^a^
X_2_^2^	0.12	0.735 ^NS^	11.70	0.007^a^	2.26	0.163 ^NS^	0.20	0.662 ^NS^
X_3_^2^	50.82	0.000^a^	15.18	0.003^a^	7.01	0.024^a^	1.38	0.267 ^NS^
X_1_X_2_	10.70	0.008^a^	346.99	0.000^a^	21.60	0.001^a^	9.79	0.011^a^
X_1_X_3_	8.64	0.015^a^	3.22	0.103 ^NS^	34.56	0.000^a^	1.80	0.209 ^NS^
X_2_X_3_	5.57	0.040^a^	0.23	0.639 ^NS^	11.45	0.007^a^	1.06	0.328 ^NS^
Lack of Fit	0.91	0.538 ^NS^	0.55	0.737 ^NS^	1.05	0.479 ^NS^	1.14	0.444 ^NS^
R^2^	0.9493		0.9975		0.9358		0.9271	
Adj- R^2^	0.9037		0.9953		0.8781		0.8614	
Pred-R^2^	0.6634		0.9905		0.7383		0.4290	

^a^*p* < 0.05; ^NS^ = not significant.

**Table 6:** Equation model for the response factor of DES extraction.

Response	Model equations	R^2^	*p*-value
Y_1_ - TAC	Y_1_ = 3.968–0.146X_1_ – 0.027X_2_ + 0.188X_3_ + 0.013X_1_^2^ + 0.018X_2_^2^ – 0.367X_3_^2^ + 0.099X_1_X_2_ – 0.089X_1_X_3_ + 0.071X_2_X_3_	0.9493	0.000
Y_2_ - TPC	Y_2_ = 21.139–1.090X_1_ + 0.649X_2_ + 3.724X_3_ + 1.962X_1_^2^ + 0.467X_2_^2^ + 0.532X_3_^2^ – 1.491X_1_X_2_ – 0.144X_1_X_3_ + 0.039X_2_X_3_	0.9975	0.000
Y_3_ - TFC	Y_3_ = 5.373–0.401X_1_ – 0.084X_2_ + 0.304X_3_ + 0.528X_1_^2^ + 0.203X_2_^2^ – 0.357X_3_^2^ – 0.368X_1_X_2_ + 0.465X_1_X_3_ + 0.268X_2_X_3_	0.9358	0.000
Y_4_ – Percent Inhibition of DPPH radical	Y_4_ = 70.885–1.111X_1_ + 0.796X_2_ + 4.591X_3_ + 3.183X_1_^2^ –+ 0.418X_2_^2^ – 1.091X_3_^2^ – 1.703X_1_X_2_ + 0.731X_1_X_3_ – 0.560X_2_X_3_	0.9271	0.000

### 3.5 Analysis of response surface

**Fig. 5:**
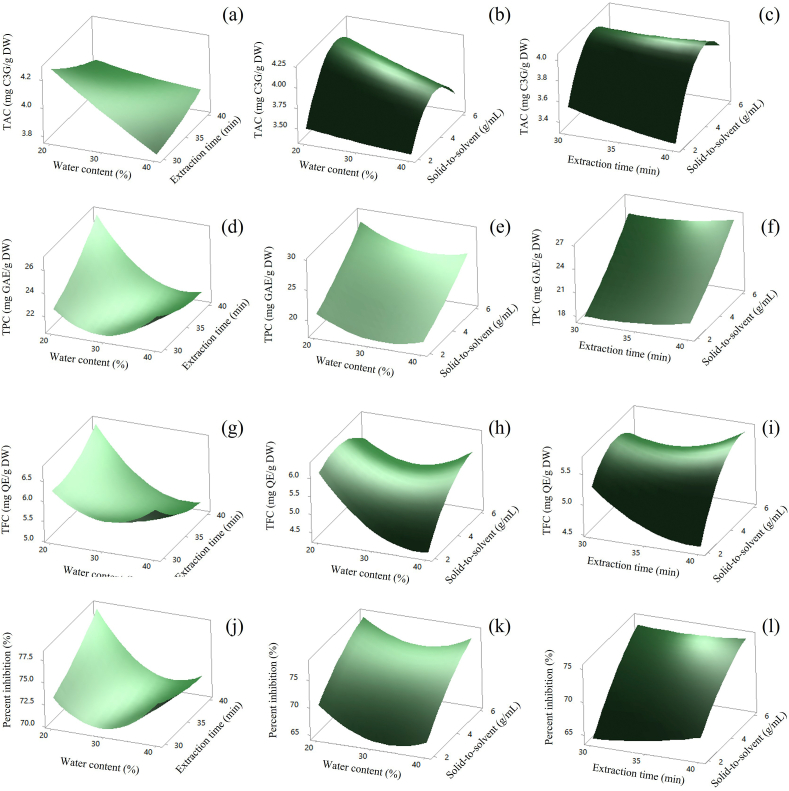
Response surface plot of DES variables on TAC (a–c), TPC (d–f), TFC (g–i) and (j–l) % inhibition.

The 3D response surface plots were generated to demonstrate the influence of parameters affecting to DES-UAE process. The effects of the independent variables (water content, extraction time, and solid-to-solvent ratio) and their interactions on TAC, TPC, TFC, and percent inhibition of DPPH radical can be seen on the three-dimensional response surface curves as shown in Fig. 5(a–l).

Fig. 5(a–c) shows the effect of water content (X_1_), extraction time (X_2_), and solid-to-solvent ratio (X_3_) on TAC. Fig. 5(a) exhibits the effect of water content (X_1_) and extraction time (X_2_) on TAC (Y_1_) at a constant rice brans-to-solvent ratio (1:4 g/mL). The highest yield of TAC was achieved (more than 4.2 mg C3G/g DW) at a water content of 20–23 % with an extraction time of 30–32.2 min. As illustrated in the results, the water content in DES mainly affected the TAC extraction yield rather than the extraction time.

The effect of water content (X_1_) and the solid-to-solvent ratio (X_3_) on TAC (Y_1_) at a constant extraction time (35 min) was illustrated in Fig. 5(b). The maximum yield of TAC was obtained (more than 4.0 mg C3G/g DW) at a water content of 20–29 % with a solid-to-solvent ratio of 1:3.5–1:6 g/mL. As illustrated in Fig. 5(b), the rice brans-to-solvent ratio had a greater impact on the TAC extraction yield than the water content in DES. In accordance with the result illustrated in Fig. 5(b), the anthocyanin content tended to increase as the rice brans-to-DES ratio increased while the water content remained constant.

Fig. 5(c) illustrates the relationship between the extraction time (X_2_) and solid-to-solvent ratio (X_3_) on TAC (Y_1_) at a constant water content (30 %). The maximum yield of TAC was acquired (more than 4.0 mg C3G/g DW) at an extraction time of 30–32.5 min with a solid-to-solvent ratio of 1:4–1.5 g/mL. The results indicated that the rice brans-to-solvent ratio showed a significant influence on TAC yield than extraction time. At constant extraction time, the TAC content increased with the ascendent of the solid-to-solvent ratio as displayed in Fig. 5(c). The interesting findings are consistent with the studies of Yanhong et al. (2020), who reported that the interaction of DES/mulberry ratio and time had an effect on anthocyanin contents [36].

The effect of water content (X_1_), extraction time (X_2_), and solid-to-solvent ratio (X_3_) on the TPC yield (Y_2_) was displayed in Fig. 5(d–f). Fig. 5(d) demonstrates the influence of water content (X_1_) and extraction time (X_2_) on TPC (Y_2_) at a constant rice brans-to-DES ratio (1:4 g/mL). The greatest yield of phenolic content was obtained (over 26.0 mg GAE/g DW) at a water content of 20–21 % with an extraction time of 38.8–40 min. Beyond this condition, the quantity of phenolic compounds tended to decrease. The effect of water content (X_1_) and the solid-to-solvent ratio (X_3_) on TPC (Y_2_) at a constant extraction time (35 min) was illustrated in Fig. 5(e). The maximum yield of TPC was achieved (more than 28.0 mg GAE/g DW) at a water content of 20–21 % with a rice brans-to-DES ratio of 1:5.8–1.6 g/mL. As illustrated in Fig. 5(e), the solid-to-solvent ratio had a greater impact on the TPC extraction yield than the water content in the DES system. In agreement with the result indicated in Fig. 5(e), the phenolic content tended to increase as the level of the solid-to-solvent ratio increased while the water content in the DES system was still constant. Fig. 5(f) illustrates the relationship between the extraction time (X_2_) and solid-to-solvent ratio (X_3_) on TPC (Y_2_) at a constant water content (30 %). The maximum yield of TPC was acquired (more than 26.0 mg GAE/g DW) at an extraction time of 38–40 min with a solid-to-solvent ratio of 1:5.8–1:6 g/mL. The results indicated that the rice brans-to-DES ratio showed a significant influence on TPC yield than extraction time. As shown in Fig. 5(f), the TPC content increased with the escalating of the solid-to-solvent ratio at constant extraction time.

The effect of water content (X_1_), extraction time (X_2_), and solid-to-solvent ratio (X_3_) on the TFC yield (Y_3_) was displayed in Fig. 5(g–i). The impact of water content (X_1_) and extraction time (X_2_) on TFC (Y_3_) at a fixed solid-to-solvent ratio (1:4 g/mL) were described in Fig. 5(g). The highest flavonoid yield (more than 6.6 mg QE/g DW) was attained at a water content of 20–21 % with an extraction duration of 38.8–40 min. The content of flavonoid compounds tended to decline beyond this point. The influence of water content (X_1_) and the solid-to-solvent ratio (X_3_) on TFC (Y_2_) at a constant extraction time (35 min) was illustrated in Fig. 5(h). The maximum yield of TPC was achieved (more than 6.0 mg QE/g DW) at a water content of 20–22 % with a solid-to-solvent ratio of 1:2–1:5.4 g/mL. The water content had a greater effect on the TFC extraction yield than the rice brans-to-DES ratio, as shown in Fig. 5(h). The association between the extraction time (X_2_) and solid-to-solvent ratio (X_3_) on TFC (Y_3_) at a constant water content (30 %) was depicted in Fig. 5(i). The highest yield of TFC was produced (more than 5.6 mg QE/g DW) at an extraction time of 30–30.6 min with a rice brans-to-DES ratio of 1:3.2–1:4.8 g/mL. The results demonstrated that the solid-to-solvent ratio had a greater impact on TFC yield than extraction duration. As shown in Fig. 5(i), the TFC yield increased with the rise of the rice brans-to-DES ratio while the extraction duration remained constant.

Fig. 5(j–l) displays the effect of water content (X_1_), extraction time (X_2_), and solid-to-solvent ratio (X_3_) on the percent inhibition of DPPH radical (Y_4_). The impact of water content (X_1_) and extraction time (X_2_) to percent inhibition (Y_4_) at a constant solid-to-solvent ratio (1:4 g/mL) were suggested in Fig. 5(j). The highest antioxidant activity (more than 78 %) was achieved at a water content of 20–23 % with an extraction time of 36.5–40 min. The percent inhibition tended to decrease beyond this point. The relationship of water content (X_1_) and the solid-to-solvent ratio (X_3_) on percent inhibition of DPPH radical (Y_4_) at a constant extraction time (35 min) was illustrated in Fig. 5(k). The results suggested that the highest percent inhibition was achieved (more than 77.5 %) at a water content of 20–20.5 % and a solid-to-solvent ratio of 1:5.6–1:6 g/mL. Beyond this point, the percentage of DPPH inhibition tended to diminish. Finally, the correlation between the extraction time (X_2_) and solid-to-solvent ratio (X_3_) on percent inhibition (Y_4_) at a constant water content (30 %) was illustrated in Fig. 5(l). The maximum percent inhibition was obtained (more than 75.0 %) at an extraction time of 30–40 min with a rice brans-to-DES ratio of 1:4.2–1:6 g/mL. The results indicated that the solid-to-DES ratio had a greater impact on percent inhibition than extraction time. As shown in Fig. 5(l), the % inhibition escalated with the rise of the solid-to-solvent ratio at steady extraction time.

### 3.6 Optimization and validation of extraction condition

According to the optimization of CCD, the optimum conditions for antioxidant extraction in Leum Pua rice bran were 20 % water content; 40 min of UAE time, and a rice bran-to-DES ratio of 1:6 g/mL. When verification investigations were performed under optimal conditions, the actual value of TAC, TPC, TFC, and percent inhibition was 4.55 ± 0.09 mg C3G/g DW, 26.49 ± 0.62 mg GAE/g DW, 6.57 ± 0.55 mg QE/g DW, and 77.83 ± 1.51 %, respectively (Table 7). The achieved results similarly showed no discernible difference between the actual and model-predicted results, which confirms the adequacy of the model [37].

**Table 7:** The optimum DES-UAE condition of the independent variable and the response of the extraction.

Responses	Optimal conditions			Predicted	Actual	Error (%)
	Water content (%)	Extraction time (min)	Solid-to-solvent ratio (g/mL)			
TAC (mg C3G/g DW)	20	40	1:6	4.05	4.55 ± 0.09	12.36
TPC (mg GAE/g DW)				30.45	26.49 ± 0.62	13.00
TFC (mg QE/g DW)				6.63	6.57 ± 0.55	0.82
Percent Inhibition of DPPH radical (%)				80.10	77.83 ± 1.51	2.83

### 3.7 Comparative study of extraction techniques and bioactive compounds in various cultivars of rice

**Fig. 6:**
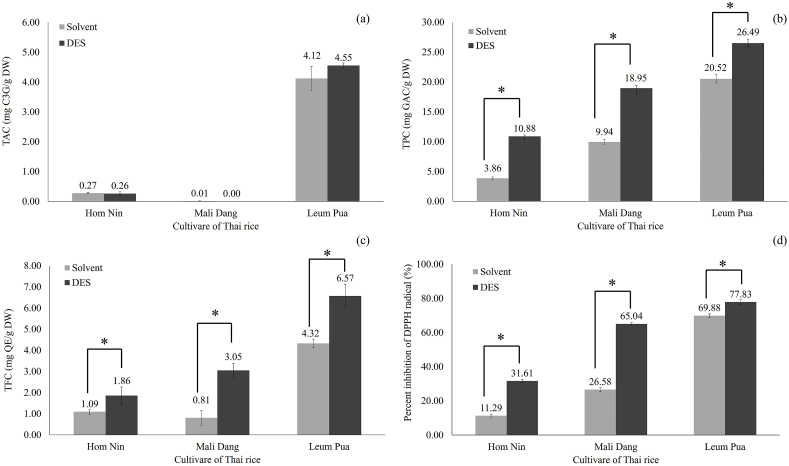
Comparison of 3 cultivars of Thai rice in optimum condition for the extraction of (a) TAC, (b) TPC, (c) TFC and (d) DPPH assay from pigmented rice bran using DES. *Level significance *p*<0.05.

The antioxidant compounds and activity obtained from the optimum condition for the extraction included the DES (Choline chloride: Ethylene glycol; 1:2) with UAE of 20 % water content, 1:6 g/mL rice brans-to-solvent ratio, and 37 kHz at 50 °C for 40 min, and conventional UAE. In Fig. 6, DES-UAE and the conventional extraction process are compared in 60 % (v/v) aqueous methanol with 0.1 % citric acid and DES. The content of TPC and TFC, ae well as DPPH assay were significantly high for DES-UAE over than conventional solvent extraction. It has been proposed that DES may provide and receive protons and electrons, forming hydrogen bonds and improving their dissolving power in the process [14]. The bioactive compounds and antioxidation activity of 3 cultivars of Thai rice (Homnin, Mali Dang, and Leum Pua) were investigated using DES-UAE (Fig. 6). The results demonstrated that Leum Pua rice contained the significant highest bioactive compounds (TAC; 4.55 ± 0.09 mg C3G/g DW, TPC; 26.49 ± 0.62 mg GAE/g DW, and TFC; 6.57 ± 0.55 mg QE/g DW, respectively) and inhibition of DPPH (77.83 ± 1.51 %). This is related to the genetic variety and growing environment of rice pertinent to a previous study [38,39]. Leum Pua demonstrated a greater level of total anthocyanin content, total phenolic content and antioxidant activity in terms of its capacity to scavenge DPPH. Hydrogen atoms are given to reactive species via hydroxyl groups, which subsequently form stable products, giving rise to antioxidant characteristics. Thus, the higher the anthocyanin content, total phenolic content, and total flavonoid content, the higher the antioxidant activity [40].

## 4 Conclusion

This study suggests that the combination of UAE and DES provides a green extraction method that is both sustainable and effective for obtaining the greatest achievable recovery of bioactive substances with strong antioxidant activity from pigmented rice bran. Environment-friendly green solvents DES (15 conditions) were used to extract antioxidant compounds (TAC, TPC, and TFC). The optimal extraction DES was found to be choline chloride:ethylene glycol (Ch:Eg) in a 1:2 ratio, which increased the extraction efficiency of antioxidant compounds. The application of central composite design to the optimization process showed 20 % water as a DES., 40 min of extraction time, and a 1:6 g/mL of rice brans-to-DES ratio. The antioxidant-rich DES extract obtained from ecologically safe and sustainable approaches has the potential for industrial applications in the food, medicine, and cosmetics sectors. For further investigations, an in-depth examination of the stability of bioactive compounds in deep eutectic solvents (DES) will be undertaken, with a focus on comprehensive comparisons.

## Data availability statement

All data generated or analyzed during this study are included in this published article.

## Additional information

No additional information is available for this paper.

## CRediT authorship contribution statement

**Pacharawan Ratanasongtham:** Writing – original draft. **Wasitthi Bunmusik:** Investigation. **Suwaporn Luangkamin:** Methodology. **Sugunya Mahatheeranont:** Supervision. **Panawan Suttiarporn:** Writing – review & editing, Conceptualization.

## Declaration of competing interest

The authors declare that they have no known competing financial interests or personal relationships that could have appeared to influence the work reported in this paper.
